# Supplementary material for: Indirect optical trapping using light driven micro-rotors for reconfigurable hydrodynamic manipulation
Source: Nat Commun. 2019 Mar 14;10:1215. doi: 10.1038/s41467-019-08968-7 (PMC6418258; doi:10.1038/s41467-019-08968-7)
Supplement: Supplementary file 1 — Supplementary Information [file 41467_2019_8968_MOESM1_ESM.pdf]

Supplementary information for:

Indirect optical trapping using light driven micro-rotors for reconfigurable hydrodynamic manipulation

Unė G. Būtaitė, Graham M. Gibson, Ying-Lung D. Ho, Mike Taverne, Jonathan M. Taylor, and David B. Phillips

Supplementary Note 1: Mathematical formulation of the hydrodynamic feedback.

The stochastic behaviour of particles in a fluid is described by the Langevin equation [1, 2]:

$$\mathbf{m}_i \odot \frac{d^2\mathbf{q}_i}{dt^2} = -\sum_{j=1}^{N} \boldsymbol{\zeta}_{ij} \frac{d\mathbf{q}_j}{dt} + \mathbf{f}_i^{\text{ext}} + \mathbf{f}_i^{\text{Brn}}, \tag{1}$$

for a system of N particles, each indexed by i or j. The equations here are expressed in a compact form in terms of generalised coordinates, encapsulating both position and orientation. Thus for a particle i the generalised mass column vector $\mathbf{m}_i = [m_i; m_i; m_i; I_i^x; I_i^y; I_i^z]$ represents mass m_i and moment of inertia I_i associated with each degree-of-freedom. Here $\odot$ represents a Hadamard multiplication (i.e. element-wise multiplication). $\mathbf{q}_i$ is a 6-element column vector encapsulating the linear and angular coordinates of particle i, i.e. $\mathbf{q}_i = [x_i; y_i; z_i; \theta_i^x; \theta_i^y; \theta_i^z]$, where x is the x-position, and θ^x is the orientation about the x-axis etc. $\mathbf{f}_i^{\text{ext}}$ is 6-element column vector denoting any external forces (first three elements) and torques (last three elements) acting on particle i, and $\mathbf{f}_i^{\text{Brn}}$ is a 6-element vector capturing the random forces and torques associated with Brownian motion. $\boldsymbol{\zeta}_{ij}$ is a 6×6 hydrodynamic friction tensor describing the coupling between all the translational and orientational degrees of freedom of particles i and j. Note for clarity: $\boldsymbol{\zeta}_{ij}$ consists of elements drawn from the full $6N \times 6N$ friction tensor $\boldsymbol{\zeta}$, describing the coupling between the entire system of N particles.

In the limit of low Reynolds number, where inertial effects are negligible compared to viscous forces, the Langevin equation can be simplified by setting the left-hand-side term to zero [3]. If we ignore the unknown Brownian term, we can rearrange Eqn. 1 to solve for a particle's velocities $\dot{\mathbf{q}}_i$ as a function of the external forces and torques acting on each particle [4]:

$$\dot{\mathbf{q}}_i = -\sum_{j=1}^{N} \boldsymbol{\mu}_{ij} \mathbf{f}_j^{\text{ext}}, \tag{2}$$

or, expanded to explicitly separate translational $\mathbf{v}$ and rotational $\boldsymbol{\omega}$ velocities:

$$\begin{bmatrix} \mathbf{v}_i \\ \boldsymbol{\omega}_i \end{bmatrix} = -\sum_{j=1}^{N} \begin{bmatrix} \boldsymbol{\mu}_{ij}^{\text{TT}} & \boldsymbol{\mu}_{ij}^{\text{TR}} \\ \boldsymbol{\mu}_{ij}^{\text{RT}} & \boldsymbol{\mu}_{ij}^{\text{RR}} \end{bmatrix} \begin{bmatrix} \mathbf{f}_j^{\text{ext}} \\ \mathbf{T}_j^{\text{ext}} \end{bmatrix}. \tag{3}$$

Here $\boldsymbol{\mu}$ is the mobility tensor, the inverse of the hydrodynamic friction tensor $\boldsymbol{\zeta}$. $\boldsymbol{\mu}^{\text{TT}}$ and $\boldsymbol{\mu}^{\text{RR}}$ are sub-tensors describing the translation-translation and rotation-rotation coupling respectively, and $\boldsymbol{\mu}^{\text{TR}}$, $\boldsymbol{\mu}^{\text{RT}}$ capture the interaction between translational and rotational degrees of freedom. $\boldsymbol{\mu}$ depends on the relative particle separations $\mathbf{r}_{ij}$ and their sizes, and can be approximated as a series expansion in $\mathbf{r}_{ij}$, as derived in [4] and given in Supplementary Note 2, for a system of multiple spheres. If necessary it can be extended further to include the presence of a planar surface, such as the bottom of the sample [5].

In our system the external forces and torques are optical in nature, generated by the optical tweezer lasers that act on the particles that compose our micro-rotors. The restoring gradient forces exerted by these optical tweezer laser beams give us direct control over these particles, allowing us to confine and manipulate them by moving the laser foci. In conjunction with our hydrodynamic control loop this in turn provides us with indirect control of the target particles.

To mathematically formulate the feedback, we first note that each of the N_{act} actuator rotors is confined by a triplet of optical traps as can be seen in Fig. 1a of the main text, such that it only has one addressable degree-of-freedom - its rotation rate about the z-axis ω^z. A rigid target particle will have N_{df} degrees of freedom, where in the most general case $N_{\text{df}} = 6$ per particle (3 translational and 3 rotational degrees of freedom), but in practice in the present paper we only control two translational degrees of freedom (x and y), and for some experiments one rotational degree-of-freedom (θ^z). Therefore, from Eqn. 3 we can derive a single matrix equation linking the target's velocity in the degrees of freedom we wish to control, $\mathbf{v}_{\text{t}}$, to the rotation rate of the rotors about their z-axes, $\boldsymbol{\omega}_{\text{r}}$:

$$\mathbf{v}_{\text{t}} = \mathbf{C}_{\text{tr}} \boldsymbol{\omega}_{\text{r}}, \tag{4}$$

where $\mathbf{v}_{\text{t}}$ is an N_{df}-element column vector containing the velocities of just the target degrees-of-freedom we wish to control, and $\boldsymbol{\omega}_{\text{r}}$ is an N_{act}-element column vector containing the required rotation rates of each actuator about their z-axis. $\mathbf{C}_{\text{tr}}$ is a $N_{\text{df}} \times N_{\text{act}}$ element matrix containing information about hydrodynamic coupling of the system and about constraints on rotor motion, which we derive below. It is the derivation and use of this equation that is central to our hydrodynamic feedback model.

For example, in a simple setup with two rotors r_1 and r_2 controlling the 2D translation of a single target t, Eqn. 4 can be expanded as:

$$\begin{bmatrix} v_{\mathrm{t}_x} \\ v_{\mathrm{t}_y} \end{bmatrix} = \begin{bmatrix} C_{\mathrm{t}_x\mathrm{r}_1} & C_{\mathrm{t}_x\mathrm{r}_2} \\ C_{\mathrm{t}_y\mathrm{r}_1} & C_{\mathrm{t}_y\mathrm{r}_2} \end{bmatrix} \begin{bmatrix} \omega_{\mathrm{r}_1} \\ \omega_{\mathrm{r}_2} \end{bmatrix}, \tag{5}$$

which we can solve by inversion to determine the required rotation rates ω_{r} that will impose the desired translation on the target particle. To derive an expression for the matrix elements in Eqn. 5 we need to consider two things: which elements of the mobility tensor are relevant, and what the constraints are on rotor motion. Both of these will depend on what type of rotors are used to perform the experiment.

If we assume that in this two rotor system, each rotor can be modelled as a single sphere that can be spun on the spot about its z-axis, and the target object is another sphere, then the matrix elements in Equation 5 are found simply by choosing the relevant elements from the mobility tensor $\boldsymbol{\mu}$ that couple rotations of the rotor spheres about their z-axis to translations of the target sphere in x and y. We note that there is an implicit assumption in doing this: we do not account for hydrodynamic coupling between the rotors themselves, i.e. we assume each rotor can freely drift in the flow-field created by the other, and there are no external optical forces constraining the location of the rotor's centre-of-mass. At first it seems that this approach is invalid as it ignores the anchoring effect of the optical tweezers which trap the rotors in 3D, holding them in the required configuration around the target. However, we note that the relaxation time of the motion of the optically trapped micro-rotors is on the order of 100 ms (estimated from the corner frequency of experimentally obtained PSD), which is considerably longer than the control-loop feedback time period of 5 ms. Therefore, over the timescale of each feedback iteration, it is reasonable to assume that the rotors are able to freely drift in each others flow. This also significantly simplifies the model of the system and so is compatible with our 200 Hz feedback rate, and we find that this approach works well in our case. In the future, it would be possible to construct a more accurate model fully taking into account the hydrodynamic interactions of all rotors with each other and the target. However, to be usefully deployed in our experiment, this would require knowledge of the optical forces felt by the rotors, necessitating real-time tracking of the position and orientation of all the rotors, significantly complicating the system. With this information, a larger matrix equation accounting for rotor coupling could be constructed and solved to calculate the required rotor motion in real-time.

The non-isotropic flow fields created by rotation of constellation rotors can also be captured by Eqn. 4. However, in this case the derivation of the matrix elements of $\mathbf{C}_{\mathrm{tr}}$ is a little more involved than above. For simplicity, we consider a setup where one rotor, consisting of three silica beads b_1, b_2 and b_3, is used to control one degree-of-freedom of the target, v_{t_x}. Here, we are only concerned with how x translation of the target couples to xy translation of each rotor bead, so we only need to consider the $\boldsymbol{\mu}^{\mathrm{TT}}$ part of the mobility tensor. To begin with, each of the three optically trapped beads has three translational degrees of freedom, so Eqn. 2 can be expanded as:

$$v_{\mathrm{t}_x} = -\left[\boldsymbol{\mu}^{\mathrm{TT}}_{\mathrm{t}_x\mathrm{b}_1}\mathbf{f}_{\mathrm{b}_1} + \boldsymbol{\mu}^{\mathrm{TT}}_{\mathrm{t}_x\mathrm{b}_2}\mathbf{f}_{\mathrm{b}_2} + \boldsymbol{\mu}^{\mathrm{TT}}_{\mathrm{t}_x\mathrm{b}_3}\mathbf{f}_{\mathrm{b}_3}\right]. \tag{6}$$

Since we are trying to determine the rotor motion required to translate the target at v_{t_x}, this is a single equation with nine unknown forces. However, in a rotor formation, beads are confined to a circular trajectory. This means that all beads in the same spinning rotor will experience the same absolute value of optical force, $F = \kappa\delta \equiv \left|\mathbf{f}_{\mathrm{b}_{1,2,3}}\right|$, where κ is trap stiffness (assuming isotropic traps) and δ is the distance between the trap and its bead. Therefore geometric considerations enable us to relate the optical forces on each bead to a single free parameter, the rotation rate of the rotor.

In 2D the direction of these vector forces can be written down relatively easily, but in 3D the derivation becomes more complicated. We have taken a general approach that is compatible with future extensions to arbitrary planes of rotation. We begin by observing that between successive updates the trap constellation is rotated by an angle of $\omega_{\mathrm{r}}\Delta t$, where Δt is the update rate and ω_{r} is the chosen angular velocity of the traps (and hence of the rotor). From geometry we find that the magnitude of the force, F, on all three beads immediately after the SLM update is $F = \omega_{\mathrm{r}}\kappa a_{\mathrm{r}}\Delta t$, where a_{r} is the rotor radius. In order to derive the direction of the force, we express the optical force acting on bead b_j as $\mathbf{f}_{\mathrm{b}_j} = \mathbf{B}_j F$ where $\mathbf{B}_j = [B_{\mathrm{b}_{jx}} B_{\mathrm{b}_{jy}} B_{\mathrm{b}_{jz}}]^{\mathrm{T}}$, and the B's are coefficients dependent on bead positions. They can be determined from the following three conditions on $\mathbf{f}_{\mathrm{b}_j}$. First, its magnitude is equal to F:

$$F^2 = F^2_{\mathrm{b}_{jx}} + F^2_{\mathrm{b}_{jy}} + F^2_{\mathrm{b}_{jz}} \qquad \Rightarrow \qquad 1 = B^2_{\mathrm{b}_{jx}} + B^2_{\mathrm{b}_{jy}} + B^2_{\mathrm{b}_{jz}} \tag{7}$$

Second, it is tangential to the ring along which the bead is moving (assuming small displacements between trap and bead), i.e., it is perpendicular to the vector, $\mathbf{a}_{\mathrm{b}_j}$, pointing from the centre of the rotor to the centre of the bead b_j. Hence, the dot product of $\mathbf{f}_{\mathrm{b}_j}$ and $\mathbf{a}_{\mathrm{b}_j}$ is zero:

$$F_{\mathrm{b}_{jx}} a_{\mathrm{b}_{jx}} + F_{\mathrm{b}_{jy}} a_{\mathrm{b}_{jy}} + F_{\mathrm{b}_{jz}} a_{\mathrm{b}_{jz}} = 0 \qquad \Rightarrow \qquad B_{\mathrm{b}_{jx}} a_{\mathrm{b}_{jx}} + B_{\mathrm{b}_{jy}} a_{\mathrm{b}_{jy}} + B_{\mathrm{b}_{jz}} a_{\mathrm{b}_{jz}} = 0 \tag{8}$$

And third, it lies in a plane perpendicular to the axis of rotation of the ring, defined by a unit vector $\hat{\mathbf{u}}_j$:

$$F_{\mathrm{b}_{jx}}\hat{u}_{jx} + F_{\mathrm{b}_{jy}}\hat{u}_{jy} + F_{\mathrm{b}_{jz}}\hat{u}_{jz} = 0 \quad \Rightarrow \quad B_{\mathrm{b}_{jx}}\hat{u}_{jx} + B_{\mathrm{b}_{jy}}\hat{u}_{jy} + B_{\mathrm{b}_{jz}}\hat{u}_{jz} = 0 \tag{9}$$

We know the location of the beads in the rotor (assuming they never lag behind their traps too much) and our traps impose the rotor's axis of rotation, therefore we know $\mathbf{a}_{\mathrm{b}_j}$ and $\hat{\mathbf{u}}_j$, and can solve Eqns. 7-9 to determine the B-coefficients for each bead in a rotor. Consequently, the number of unknowns in Eqn. 6 is reduced to one and we can rewrite it to fit the form of Eqn. 4:

$$\begin{aligned} v_{\mathrm{t}_x} &= -\left[\boldsymbol{\mu}_{\mathrm{t}_x\mathrm{b}_1}\mathbf{B}_{\mathrm{b}_1} + \boldsymbol{\mu}_{\mathrm{t}_x\mathrm{b}_2}\mathbf{B}_{\mathrm{b}_2} + \boldsymbol{\mu}_{\mathrm{t}_x\mathrm{b}_3}\mathbf{B}_{\mathrm{b}_3}\right] F \\ &= -\sum_{j=1}^{3} \boldsymbol{\mu}_{\mathrm{t}_x\mathrm{b}_j}\mathbf{B}_j \ \kappa a_{\mathrm{r}} \Delta t \omega_{\mathrm{r}} \\ &= C_{\mathrm{t}_x r}\omega_{\mathrm{r}}. \end{aligned} \tag{10}$$

For clarity we now demonstrate the equivalent derivation for the case of a single wheel-like micro-rotor controlling target's velocity v_{t_x}. These micro-rotors are set into motion by applying a torque $\mathbf{T}_{\mathrm{r}}$ via translation of the handle spheres, so now we need to consider the $\boldsymbol{\mu}^{\mathrm{TR}}$ part of the mobility tensor. The equivalent to Eqn. 6 is now:

$$v_{\mathrm{t}_x} = -\boldsymbol{\mu}^{\mathrm{TR}}_{\mathrm{t}_x r}\mathbf{T}_{\mathrm{r}}. \tag{11}$$

We can write the torque as $\mathbf{T}_{\mathrm{r}} = \hat{\mathbf{b}}_{\mathrm{r}} T_{\mathrm{r}}$, in terms of the torque magnitude T_{r} and a unit vector $\hat{\mathbf{b}}_{\mathrm{r}}$ representing the axis of rotation of the micro-rotor. Since mobility tensors for structures as complicated as the DLR rotors are not readily available, in order to relate the torque to the rotation rate we model the rotor as a single spinning spherical particle. This enables us to use the known relation $T = 8\pi\eta a^3\omega_{\mathrm{r}}$, where η is viscosity of the surrounding medium and a is the radius of the particle. Now we expand Eqn. 4 as before, this time yielding:

$$\begin{aligned} v_{\mathrm{t}_x} &= -\boldsymbol{\mu}^{\mathrm{TR}}_{\mathrm{t}_x r}\mathbf{B}_{\mathrm{r}} 8\pi\eta a_{\mathrm{r}}^3\omega_{\mathrm{r}} \\ &= C_{\mathrm{t}_x r} R_{\mathrm{r}}. \end{aligned} \tag{12}$$

This now has given us the required result - the coupled relationship between the velocity of the target bead and the rotation rates of the actuators. Equivalents to Eqns. 10 and 12 can be derived for any number of rotors and any number of target degrees of freedom. Note though, that this system of equations may have three distinct behaviors. It can be overdetermined if we do not have enough rotors, such as if we try to control target's translation in x and y with a single rotor; in this case, the system will only have a solution in certain special-case configurations. If the number of actuators is the same as the number of degrees of freedom of the target, $\mathbf{C}_{\mathrm{tr}}$ will be a square matrix - the system will have a unique solution as long as $\mathbf{C}_{\mathrm{tr}}$ is invertible, i.e., there could still be some target-rotor configurations for which there is no solution. And finally, the system can have an excess of rotors and be underdetermined. In this case, as long as the equations are consistent, there will be infinitely many solutions. See Supplementary Note 4 for a description of what we do in this case.

Once the elements of $\mathbf{C}_{\mathrm{tr}}$ in Eqn. 4 are known, we input our required target velocity, which is determined as the required velocity to push the target from its most recent measured location to our desired location in one feedback loop iteration time (i.e. 5 ms), and solve Eqn. 4 for the rotor rotation rates. These rotation rates may exceed the maximum rotation rates we can achieve in our system before the rotors are lost from the optical traps. If the highest required rotation rate exceeds our experimental limit (typically $\pi/2\,\mathrm{rad\,s^{-1}}$) all rotation rates are scaled down so that the highest rate equals our experimental limit. This method ensures that the direction the target is pushed in is preserved, but reduces the magnitude of the flow across the target. See conclusions of the main paper for a discussion of other limitations of the system.

Supplementary Note 2: Full equations for the mobility tensor.

Given below are the equations for mobility tensor elements derived in [4] for a system of N spherical particles, indexed by i or j and with position vectors $\mathbf{x}_i$ or $\mathbf{x}_j$:

$$6\pi\eta a_i \boldsymbol{\mu}_{ij}^{\mathrm{TT}} = \mathbf{I}_3\delta_{ij} + \frac{3a_i}{4r_{ij}}(\mathbf{I}_3 + \hat{\mathbf{r}}_{ij}\hat{\mathbf{r}}_{ij})(1-\delta_{ij}) \tag{13}$$

$$12\pi\eta a_i^2 \boldsymbol{\mu}_{ij}^{\mathrm{RT}} = -\frac{3a_i^2}{2r_{ij}^2}\boldsymbol{\epsilon}\cdot\hat{\mathbf{r}}_{ij}(1-\delta_{ij}) \tag{14}$$

$$8\pi\eta a_i^2 a_j \boldsymbol{\mu}_{ij}^{\mathrm{RR}} = \mathbf{I}_3\delta_{ij} + \frac{3a_i^2 a_j}{2r_{ij}^3}(\hat{\mathbf{r}}_{ij}\hat{\mathbf{r}}_{ij} - \frac{\mathbf{I}_3}{3})(1-\delta_{ij}) \tag{15}$$

$\mathbf{I}_3$ is a 3 x 3 identity matrix, $\boldsymbol{\epsilon}$ is the Levi-Civita tensor defined below, δ_{ij} is the Kronecker delta, $\hat{\mathbf{r}}_{ij}$ is a unit vector in the direction of the bead separation vector, $\mathbf{r}_{ij} = \mathbf{x}_j - \mathbf{x}_i$, with magnitude r_{ij}, $a_{i,j}$ is particle radius, and η is the surrounding fluid viscosity. The components of the mobility tensor obey the following symmetry relations: $\boldsymbol{\mu}_{ij}^{\mathrm{TT}} = [\boldsymbol{\mu}_{ji}^{\mathrm{TT}}]^{\mathrm{tr}}$, $\boldsymbol{\mu}_{ij}^{\mathrm{RR}} = [\boldsymbol{\mu}_{ji}^{\mathrm{RR}}]^{\mathrm{tr}}$ and $\boldsymbol{\mu}_{ij}^{\mathrm{RT}} = [\boldsymbol{\mu}_{ji}^{\mathrm{TR}}]^{\mathrm{tr}}$, where the superscript tr denotes a transpose. In three dimensions k, l, m the Levi-Civita tensor $\boldsymbol{\epsilon}$ is defined as follows:

$$\epsilon_{k,l,m} = \begin{cases} +1 & \text{if } (k,l,m) \text{ is } (1,2,3),\ (2,3,1) \text{ or } (3,1,2) \\ -1 & \text{if } (k,l,m) \text{ is } (3,2,1),\ (2,1,3) \text{ or } (1,3,2) \\ 0 & \text{if } k=l, \text{ or } k=m, \text{ or } l=m. \end{cases} \tag{16}$$

Supplementary Note 3: Calculating fluid flow-fields.

Flow fields around translating and/or rotating particles can be estimated using Eqns. 3 and 13-15 in the limit $a_i \to 0$. In practice this is performed by defining a 2D grid and placing a zero size tracker particle on each point of the grid. The relevant mobility tensor terms are then calculated and velocity of the tracer particle determined. Note that when calculating fluid velocities we considered additional terms in the expansion for $\boldsymbol{\mu}_{ij}^{\mathrm{TT}}$:

$$6\pi\eta a_i \boldsymbol{\mu}_{ij}^{\mathrm{TT}} = \mathbf{I}_3\delta_{ij} + \left[\frac{3a_i}{4r_{ij}}(\mathbf{I}_3 + \hat{\mathbf{r}}_{ij}\hat{\mathbf{r}}_{ij}) - \frac{3a_i}{4r_{ij}^3}(a_i^2 + a_j^2)(\hat{\mathbf{r}}_{ij}\hat{\mathbf{r}}_{ij} - \frac{\mathbf{I}_3}{3})\right](1-\delta_{ij}). \tag{17}$$

This expansion is known as the Rotne-Prager tensor. As noted in Supplementary Note 1 the shape of DLW micro-rotors is too complicated to simulate their exact hydrodynamic behaviour in our developed model, so we treat them as spherical beads. Since we cannot place tracker particles inside spherical rotors, we assumed a $1/r^2$ fluid velocity fall-off from the inside edge of the rotor disk, to plot the flow-fields inside the micro-rotors seen in Fig. 1(c-e).

Supplementary Note 4: Solving an underdetermined control system.

As noted above, Eqn. 4 may represent an underdetermined system of equations. An underdetermined system will have no solution if the equations are inconsistent, i.e., if the rank of the augmented matrix $[\mathbf{C}_{\mathrm{tr}}\ \ \dot{\mathbf{q}}_t]$ is greater than the rank of the coefficient matrix $\mathbf{C}_{\mathrm{tr}}$ (Rouche-Capelli theorem). An underdetermined system will usually have a set of infinite solutions, and solver functions such as the Solve Linear Equations.vi in LabVIEW will find one of the set of solutions. We found, however, that common implementations often yield a solution in which one of the rotors is stationary. Since the performance of our system is normally limited by the maximum possible rotation rate of the rotors, we define the optimum solution to be the one in which the rotation rate of the fastest rotor is minimised. Since at this point our system of equations is computationally fast to solve, we identify this solution by a simple trial-and-error method in which we input a range of different fixed rotation rates for one of the rotors, and for each fixed input, compute the accompanying trial solutions for the rest of the rotors. Our estimate is refined with further rounds of trial solutions in the vicinity of the best solution of the previous iteration. The algorithm terminates if the solution is no longer changing or after 11 iterations.

Supplementary Note 5: Clamping of a small particle and translational speed of the target.

Shown in Supplementary Figure 1 are results for hydrodynamic clamping of a 2.5 µm radius silica bead, with the standard deviation $\sigma_{\text{mean}} = 186\,\text{nm}$.

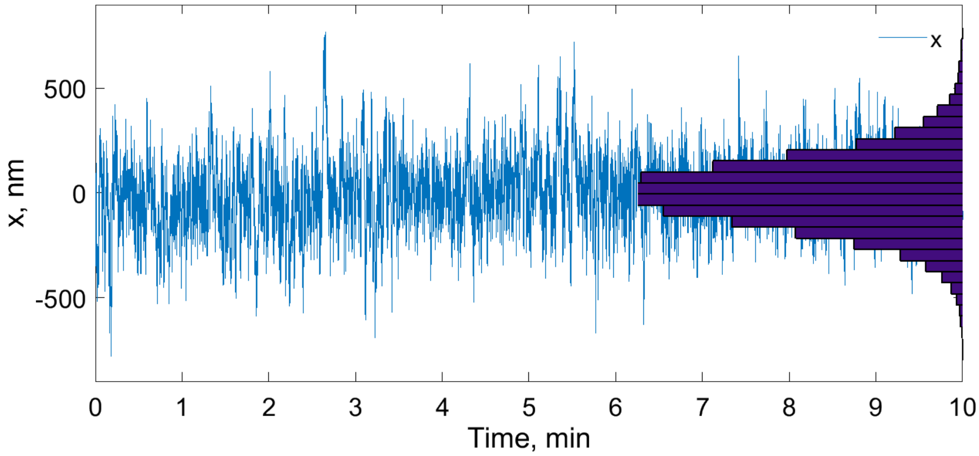

Supplementary Figure 1: Trace and 1D histogram of the x-coordinate of a hydrodynamically clamped 2.5 µm radius silica bead, using 3-bead constellation rotors, positioned 22 µm away from the target.

Presented on the LHS of Supplementary Figure 2 are the results for the speed of the target following the trajectory shown in Fig. 3a of the main text and in Supplementary Video 2, as a function of rotor-target separation, d.

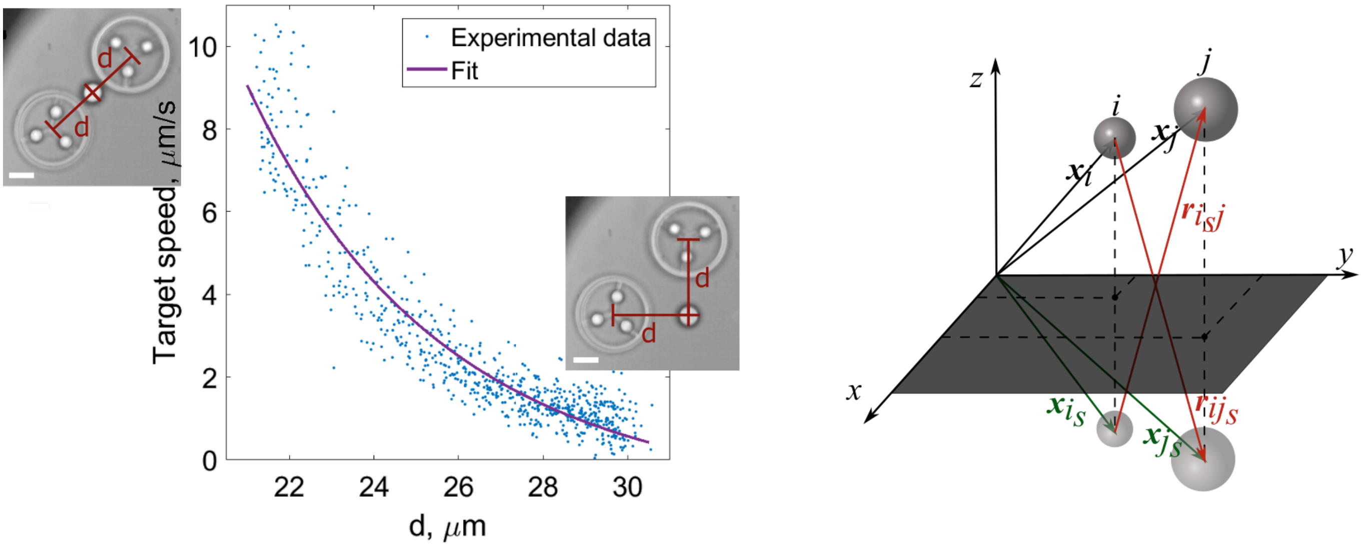

Supplementary Figure 2: Translational speed of a 5 µm radius target particle as a function of target distance from the rotors d (left) and a schematic illustrating the notation conventions used in the mathematical description (right). The white scale bar represents the distance of 10 µm.

To determine the fit, we begin by referring to Eqn. 3 in Supplementary Note 1, and noting that for the case of a single target bead t located a distance d away from a spinning micro-rotor r, exposed to optical torque $\mathbf{T}_\text{r}$, target's velocity will be given by:

$$\mathbf{v}_\text{t} = -\boldsymbol{\mu}_{\text{tr}}^{\text{TR}}\mathbf{T}_\text{r}. \tag{18}$$

From Eqn. 14 in Supplementary Note 2 we know that $\boldsymbol{\mu}_{\text{tr}}^{\text{RT}}$ has a $1/d^2$ dependency. However, to obtain an accurate fit, we must take into account the bottom surface of the sample, near which the target bead is moving. This can be

done by including additional terms in the expansion for $\boldsymbol{\mu}_{ij}^{\mathrm{TR}}$, as derived in [5]. For two particles i and j we now have

$$12\pi\eta a_i^2 \boldsymbol{\mu}_{ij}^{\mathrm{TR}} = -\frac{3a_i^2}{2r_{ij}^2}\boldsymbol{\epsilon}\cdot\hat{\mathbf{r}}_{ij}(1-\delta_{ij}) + \frac{3a_i^2}{2r_{ij_{\mathrm{s}}}^2}\boldsymbol{\epsilon}\cdot\hat{\mathbf{r}}_{ij_{\mathrm{s}}}, \tag{19}$$

where $r_{ij_{\mathrm{s}}} \equiv (r_{ij}^2 + 4l_i l_j)^{1/2}$ is the distance between particle i and the mirror image of particle j, see RHS of the figure, and l_i and l_j are the distances from the wall to the particles. In our case $l_i = l_j = l$ and, after simplifying, Eqn. 18 has the following dependency:

$$v_{\mathrm{t}} \propto \frac{c_1}{d^2} - \frac{c_2}{d^2 + 4l^2}, \tag{20}$$

where we have introduced scaling factors c_1 and c_2. Equation 18 is fitted to the experimental data in Supplementary Figure 2 (purple line) with $c_1 = 37\,440\,\mu\mathrm{m}^2\,\mathrm{s}^{-1}$ and $c_2 = 41\,030\,\mu\mathrm{m}^2\,\mathrm{s}^{-1}$. To obtain the fit we assume the distance form the surface l to be equal to the radius of the target particle, $5\,\mu\mathrm{m}$. We also note that Eqn. 19 dictates that velocity of a target particle being translated by a spinning actuator is nominally independent of the target's size a_{t}.

Supplementary Note 6: Hydrodynamic clamping of two target particles.

Shown in Supplementary Figure 3 are the setup and results of hydrodynamic manipulation of 2 target particles. We obtained clamping results with standard deviation of $\sigma_{\text{mean}} = 120\,\text{nm}$. We believe that the increase in σ_{mean}, compared to that of clamping a single bead ($\sigma_{\text{mean}} = 79\,\text{nm}$), is due to several factors. The five micro-rotors are positioned further away from the targets, decreasing their hydrodynamic influence on the beads. Furthermore, the trapping laser power is now split between more rotors, making their optical trapping less efficient and their rotation rates lower. In addition, the structure of flow fields needed to control multiple target particles is significantly more complex, enforcing lower fluid velocities, as discussed in the conclusions of the main text.

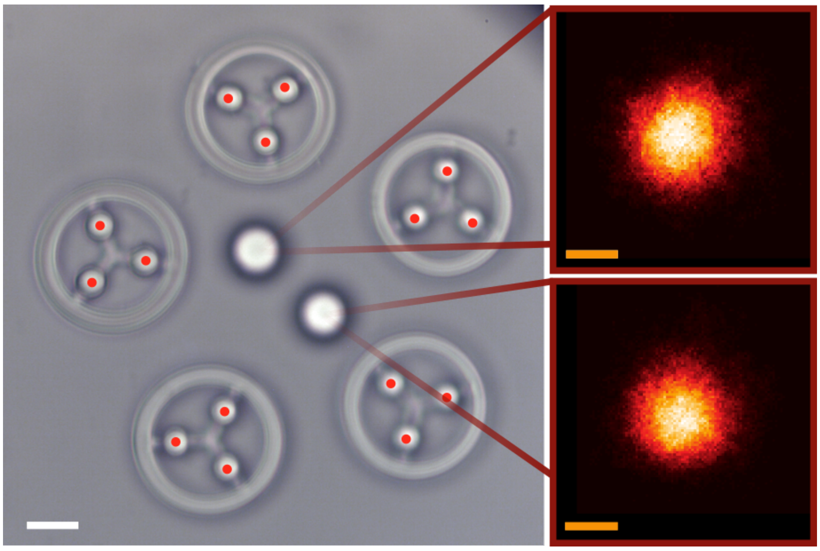

Supplementary Figure 3: Hydrodynamic clamping of two 5 µm radius target particles using five micro-rotors, each of 22 µm in diameter, positioned 28 µm away from the centre of mass of the two targets. The white scale bar indicates the distance of 10 µm and the orange scale bars - that of 20 nm.

Shown in Supplementary Figure 4 is the time trace of two hydrodynamically manipulated target particles (see Supplementary Video 9).

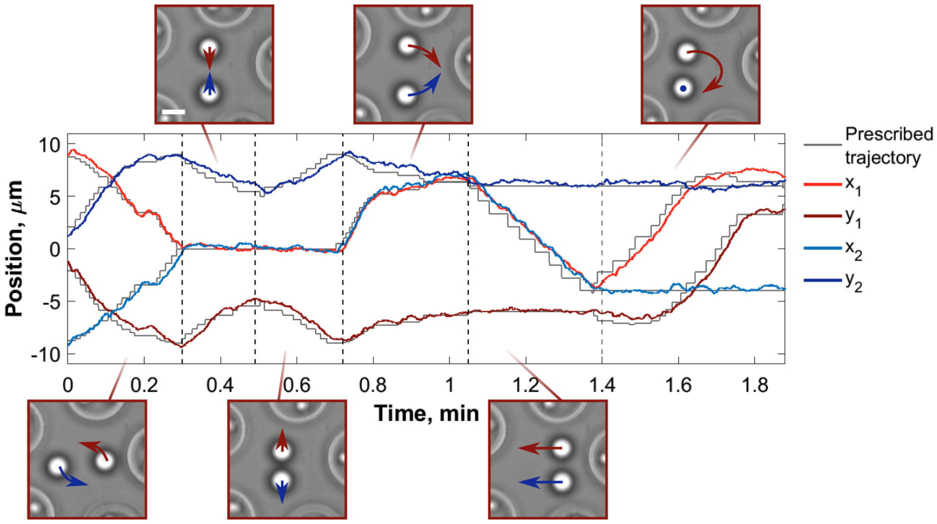

Supplementary Figure 4: The time trace of two 5 µm radii target particles as they are hydrodynamically manipulated to follow a prescribed trajectory. The 22 µm diameter micro-rotors are positioned on a ring of 28 µm radius. Here the white scale bar represents the distance of 10 µm.

Supplementary Note 7: Experimental setup.

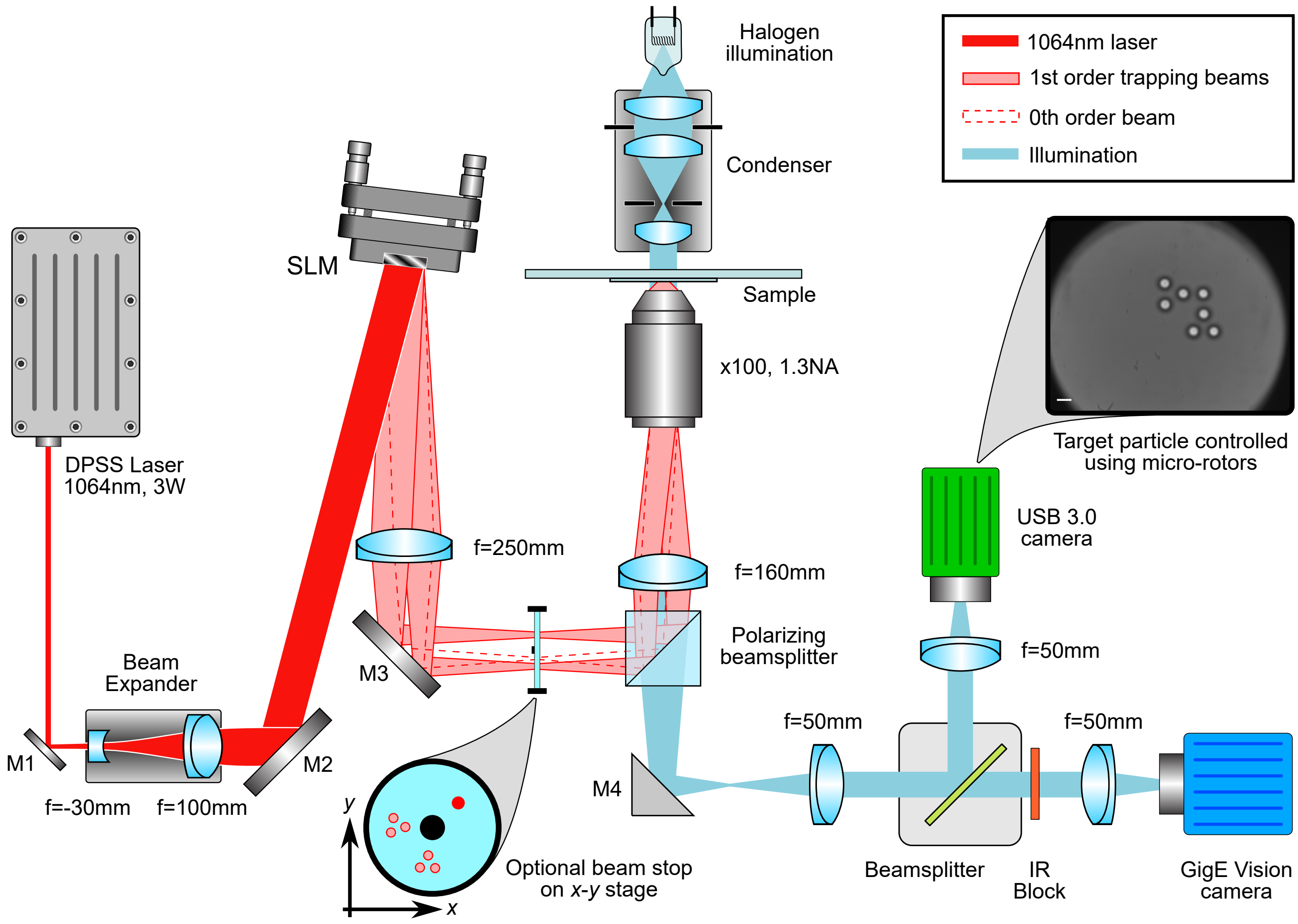

Supplementary Figure 5: Experimental setup.

A continuous-wave diode-pumped solid-state (DPSS) laser (Laser Quantum: VentusIR, 3W) is used to generate a beam of wavelength 1064 nm. This beam is then expanded and directed to slightly overfill a reflective spatial light modulator (SLM) (Boulder Nonlinear Systems: XY-series, 512× 512 resolution). The SLM imprints a reconfigurable phase pattern onto the beam, enabling generation of multiple optical traps in the sample. The field at the SLM is re-imaged, using $4f$ relay optics, onto the back aperture of a high NA oil-immersion objective lens (Nikon Plan Fluor: 100×; 1.3NA). The microscope lens focuses the light into the sample, forming the optical traps.

Because the SLM is only capable of phase modulation, there will be low-intensity ghost orders present in the sample in addition to the main optical traps. To make sure that this unwanted light does not interfere with the target particles, in some experiments we have used a translatable beam stop. Simple in design - a glass slide with a non-transparent spot - this beam block was placed in the image plane of the focused beams in the sample, and positioned so that any unwanted light destined for the region of the target particles was blocked, see also Supplementary Figure 6.

To image the sample, it was illuminated with a halogen illumination module (Zeiss Axiovert: 100W) fitted with a 0.55 numerical aperture (NA) condenser. The illuminating light was collected using the same microscope objective lens as used to focus the optical traps, and then directed onto a beam-splitter. The transmitted light then goes through an IR filter to block any residual trapping laser light from reaching camera 1 (GigE Vision, Teledyne DALSA Genie: HM1024), which is used for high frame rate particle tracking. The light reflected from the beam-splitter is focused onto camera 2 (USB 3.0, JAI GO: JAI GO-5000M-USB), used for observing and video recording the experiments at the full field-of-view of the microscope objective (the scale bar in the image represents a distance of 10 µm).

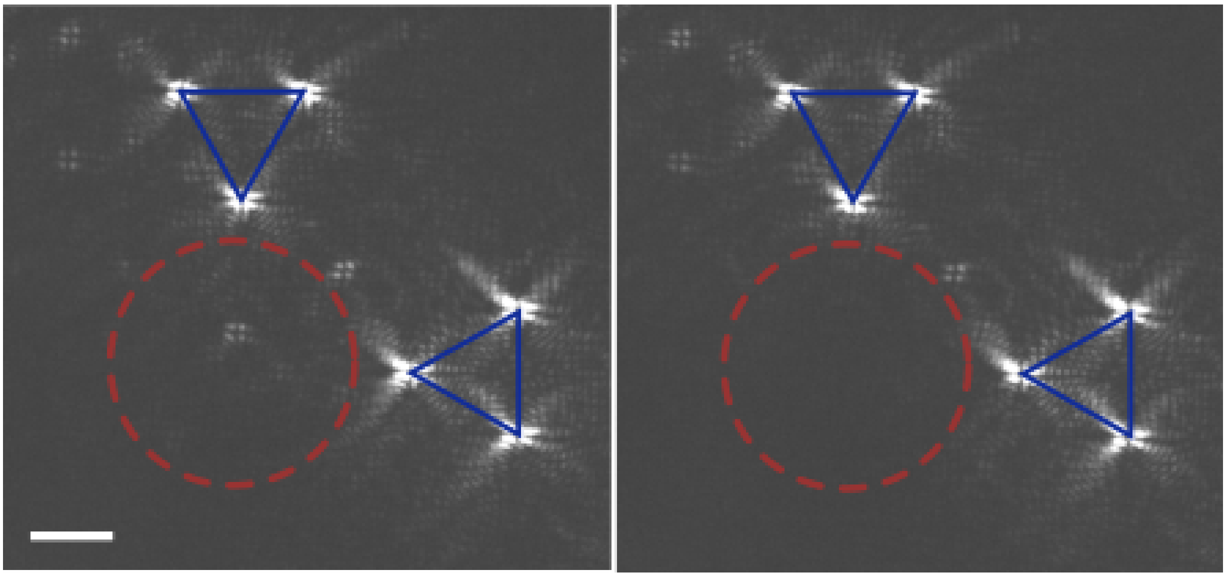

Supplementary Figure 6: Blocking out light from the target area. This figure shows reflections of the optical trapping light from a sample glass slide. Shown here is the arrangement used to manipulate one target with two rotors. Each micro-rotor is held in three optical traps, connected with blue lines. As can be seen in the area of the red circle on the LHS of the figure, unwanted ghost orders are formed in the target area. Note that here more light was intentionally directed into the ghost orders, to make them visible to the naked eye. To ensure that the target is exposed to as little light as possible, in some experiments we placed a circular beam block in the conjugate image plane (as shown in Supplementary Figure 5). The effect is shown on the RHS of the figure, with a clean target area. Here the white scale bar represents the distance of 10 µm.

Supplementary references

[1] Ermak, D. L. & McCammon, J. Brownian dynamics with hydrodynamic interactions. *The Journal of chemical physics* **69**, 1352–1360 (1978).

[2] Coffey, W., Kalmykov, Y. P. & Titov, S. Langevin equation method for the rotational brownian motion and orientational relaxation in liquids. *Journal of Physics A: Mathematical and General* **35**, 6789 (2002).

[3] Purcell, E. M. Life at low reynolds number. *American journal of physics* **45**, 3–11 (1977).

[4] Mazur, P. & Van Saarloos, W. Many-sphere hydrodynamic interactions and mobilities in a suspension. *Physica A: Statistical Mechanics and its Applications* **115**, 21–57 (1982).

[5] Beenakker, C., Van Saarloos, W. & Mazur, P. Many-sphere hydrodynamic interactions: Iii. the influence of a plane wall. *Physica A: Statistical Mechanics and its Applications* **127**, 451–472 (1984).
