# Supplementary material for: Indirect optical trapping using light driven micro-rotors for reconfigurable hydrodynamic manipulation
Source: Nat Commun. 2019 Mar 14;10:1215. doi: 10.1038/s41467-019-08968-7 (PMC6418258; doi:10.1038/s41467-019-08968-7)
Supplement: Supplementary file 12 — Description of Additional Supplementary Files [file 41467_2019_8968_MOESM12_ESM.docx]

**Title:** Supplementary Video 1: Hydrodynamic clamp of a single target bead using micro-rotors.
**Description:** This video shows data presented in Fig. 2a. The orange dot indicates the desired location of the target.

**Title:** Supplementary Video 2: Translating the target with stationary rotors.
**Description:** This video shows data presented in Fig. 3a. The orange dot indicates the location to which the feedback is told to translate the target to, and the blue dots indicate the position of the target up to 5 s in the past.

**Title:** Supplementary Video 3: Translating the target with mobile rotors.
**Description:** This video shows a target being translated with the help of two micro-rotors, which are programmed to follow the target. The orange dot indicates the location to which the feedback is told to translate the target to, and the blue dots indicate the position of the target up to 2 s in the past.

**Title:** Supplementary Video 4: Translating the target by moving the sample.
**Description:** This video shows the experiment in Fig. 3c. The blue and grey dots indicate the position of the target while the sample is being translated across the eld of view at 1 µm s−1 .

**Title:** Supplementary Video 5: Hydrodynamic clamp of a single target bead using constellation bead rotors.
**Description:** This video shows data in Fig. 4d. The orange dot indicates the desired location of the target.

**Title:** Supplementary Video 6: Hydrodynamic clamp of a chromium particle using constellation bead rotors.
**Description:** The first section of this video shows a piece of chromium exposed to an optical trap (red circle) and being pushed away from it. The second section shows a piece of chromium being clamped hydrodynamically as in data shown in Fig. 4g. The orange dot indicates the desired location of the target.

**Title:** Supplementary Video 7: Hydrodynamic manipulation of a yeast cell using constellation yeast rotors.
**Description:** This video shows data in Fig. 4i. The orange circle indicates the trajectory the target is meant to follow, and the blue dot shows the location of the target yeast.

**Title:** Supplementary Video 8: Hydrodynamic clamping of translation and orientation of a yeast cell. **Description:** This video shows data in Fig. 4j. The orange cross-hair indicates the desired location and orientation of the target, and the blue cross-hair shows the actual location and orientation of the target yeast. Supplementary Video 9: Hydrodynamic manipulation of multiple particles. This video shows the experiment in Fig. 5f and Supplementary Figure 4. The orange lines show the trajectory the targets are prescribed to follow up to 10s in the future, and the blue dots indicate location of the targets, up to 5 s in the past.
